# Eco-Innovation Analyses in the Management of Drinking Water Provided by the Main Suppliers in Romania

**DOI:** 10.3390/ijerph18126232

**Published:** 2021-06-09

**Authors:** Oana-Adriana Crișan, Corina Bîrleanu, Horea-George Crișan, Marius Pustan, Violeta Merie, Florina Șerdean

**Affiliations:** 1 Micro-Nano Systems Laboratory, Technical University of Cluj-Napoca, 400641 Cluj-Napoca, Romania; 2 Mechanical Systems Engineering Department, Machine Building Faculty, Technical University of Cluj-Napoca, 400641 Cluj-Napoca, Romania; Corina.Barleanu@omt.utcluj.ro (C.B.); Horea.Crisan@omt.utcluj.ro (H.-G.C.); Marius.Pustan@omt.utcluj.ro (M.P.); Florina.Rusu@omt.utcluj.ro (F.Ș.); 3 Materials Science and Engineering Department, Materials and Environmental Engineering Faculty, Technical University of Cluj-Napoca, 400641 Cluj-Napoca, Romania; Violeta.Merie@stm.utcluj.ro

**Keywords:** eco-innovation, drinking water management, sustainable consumption, economic operators in water supply, development regions of Romania

## Abstract

The transition to a circular economy with an emphasis on eco-innovation is just beginning both in Europe and in Romania, as a member country of the European Union. The whole economic system in which people operate must be circular, which means that it must eliminate conflicting aspects related to regulation, collaboration, governance, supply chain dynamics, and data transparency. However, the barriers to the transition to a circular economy are substantial, and it is up to states to work together to find innovative solutions to society’s problems. This article focuses on aspects related to eco-innovation in the field of drinking water quality in all administrative regions of Romania. In this regard, a study was undertaken, and the main objective pursued in was to identify and highlight the degree of eco-innovation of drinking water suppliers in seven underdeveloped regions of Romania. Starting from an analysis of the water management framework through the OECD Principles on Water Governance, it was possible to develop a study on drinking water supply companies in Romania. This study was performed based on specific indicators grouped by categories, and it aimed in two directions: on the one hand, the identification of drinking water distributors with a high degree of eco-innovation, which leads to quality certification of the drinking water supplied and which has the impact of encouraging household consumers to mainly use this type of supplied water to the detriment of PET bottled water (which has well-known major disadvantages in relation to environmental pollution and user health); on the other hand, the identification of drinking water supply companies with a low degree of eco-innovation, which is proof of the need for mandatory measures to improve drinking water quality, measures that can be taken at the supplier level but especially with support from the administrative and political environment.

## 1. Introduction

Our economies have developed a “take–make–consume–dispose” growth pattern—a linear model that is based on the assumption that resources are abundant, available, and easily accessible and that it is cheap to eliminate their waste and byproducts from the circuit. Experience has shown that such a direction is wrong and that greater and more sustainable performance can be achieved if resources are used in a more efficient way. In this regard, architects, chemists, engineers, as well as other experts have observed that the global economy can indeed be run in a different way. Like specialists, today’s leaders work together to think about this issue in a holistic manner [1,2].

As Sariatli also said, the intrinsic mechanics of the linear economy (by counting on the wasteful take—make—dispose flow) is detrimental to the environment and cannot supply the growing populace of our planet with essential services, which naturally results in strained profitability [3,4].

The water innovation sector takes part in the global concept of the circular economy. Starting from the 5 Rs principle (Refuse, Reduce, Reuse, Repurpose, Recycle), growing the eco-innovation level amongst the drinkable water providers produces positive results. Increasing the extent of providing high-quality water and increasing the number of consumers through applied eco-innovation methods, can also lead to a reduction in the quantity of bottled water purchased and an implicit reduction in the costs of acquiring drinkable water.

However, the most important aspect is the possibility of reducing the PET (polyethylene terephthalate) waste from water bottle packaging, which is well known to be a significant source of pollution.

However, water in general and drinking water in particular is considered to be an essential resource for the existence of humans and other living beings. Even if water is an infinite resource for use on the European continent, signals on long-term climate change and hydrological assessments, including changes in population dynamics, indicate a decrease in per capita renewable water resources.

According to the European Commission, “eco-innovation refers to all forms of innovation—technological and non-technological—that create opportunities for the development of economic activities and have a positive effect on the environment by preventing or reducing the impact of these activities, or by optimizing resource use” [5]. According to the European Eco-Innovation Observatory, this involves “introducing or modifying any product, service, process, organizational change or marketing solution that contributes to reducing resource use and reducing the release of toxic substances throughout the life cycle” [6].

It can be said that eco-innovation of economic operators in the supply of drinking water could be a first step in the proper management of this sector, causing the population to move towards a sustainable mode of consumption, eliminating consumption options that are less friendly to the environment [7].

In other words, it can be said that eco-innovation methods are less harmful to the environment than the regular less ecologically oriented methods that are currently used at a very large scale [8]. Eco-innovation has been implemented by the majority of the European states. Nowadays, there are six states that have successfully implemented this good practice model: Luxembourg, Denmark, Finland, Sweden, Austria, and Germany [9]. Regarding compliance with eco-innovation indicators, in relation to the average of the European Union member states, a 20% decrease in compliance was registered in the last year compared with the first year of the analyzed time period. This result raises another great alarm in the sense of identifying an immediate need to take corrective measures, particularly in the Romanian political environment [10].

Starting from the above-mentioned environmental aspects, the present research paper aims to present results related to the qualitative aspects of drinking water in close relationship with aspects of its eco-innovation, based on nine indicators belonging to the following six groups:

- Inputs in the field of eco-innovation;
- Eco-innovation activities;
- Eco-innovation results;
- A specific indicator regarding the requirements of the performed study;
- Socio-economic results;
- Resources efficiency.

The indicators from these groups were derived from the General Key Indicators adopted by the EU through the monitoring framework for the circular economy, and they can be applied to all water supply companies in the underdeveloped regions of Romania.

For the beginning, a framework analysis of eco-innovation in the water management sector was conducted, which first took into account the Organization for Economic Co-operation and Development (OECD) principles on water governance and existing legislation and directives, which reflect the main objectives to be achieved in this sector at the European level in the coming decades. Furthermore, in accordance with the proposed objectives, the analysis was detailed in particular for Romania, the country that is the subject of the case study in this paper.

The analysis highlights Romania’s level of eco-innovation in relation to other EU member states from the perspective of general eco-innovation indicators, as well as highlighting approaches to improving performance in water management from the perspective of eco-innovation, especially aimed at drinking water consumed by the population.

Within the same analysis, by identifying the possibilities for developing economic activities towards reducing the resources used and reducing the negative impact on the health of the population and the surrounding environment in the process of drinking water production and distribution, the development of eco-innovation in water management was further investigated from the perspective of sustainability. The analysis was the basis of the study conducted to determine the possibility of implementing eco-innovative measures in the drinking water management sector in Romania from the perspective of sustainability, which was oriented on the circular economy model.

Within the description of the materials and the method used in the study elaborated here, first the general indicators that are suitable to be used are identified from among those that have been established at EU level, then the activities needed to achieve the proposed objectives are described, followed by identification of economic operators for the supply of drinking water in the underdeveloped regions of Romania, an explanation of the indicators and sub-indicators used and the impact on the results of the study, and a presentation of the case study itself, which was carried out with the aim of evaluating the regional operators—drinkable water suppliers—in accordance with the specified premises.

Using a pre-established scoring system on the level of implementation regarding the analyzed indicators, a classification of drinking water distribution companies was performed, in accordance with their performance in the field of sustainable eco-innovation in drinking water management. The obtained results were interpreted from a statistical point of view in order to identify the areas in need of immediate action or measures that could soon improve a supplier’s activity from a technological, economic, resource management, and distribution network perspective in order to provide high-quality drinking water and to make a major contribution to consumers’ decisions to give up the consumption of drinking water bottled in PET packaging. These measures are extremely timely as they bring only benefits to consumer health, the environment, and the economy; however, their implementation depends on several actors who have to work together, the largest being the actual operator who provides drinking water in a particular area but not least the political entities that administer locally or regionally or that govern at the national level.

Therefore, the importance and necessity of this theme addresses the finite resource of water and its continuing decline in drinkable quality, which puts pressure as well on researchers to find new approaches to water production and consumption.

## 2. Analytical Framework of the Concept of Eco-Innovation in the Water Management Sector

### 2.1. Water Management Framework

According to the Organization for Economic Co-operation and Development, global pressures on water and related sectors around the world require action [11]:

- Accessible and high-quality fresh water is a limited and highly variable resource. OECD projections show that 40% of the world’s population currently lives in river basins, which is why water demand will increase up to 55% by 2050.
- By 2050, 240 million people are expected to be without access to clean water and 1.4 billion are expected to be without access to basic sanitation.
- Significant investment is needed to renew and modernize infrastructure, estimated at $ 6.7 trillion by 2050 for water supply and sanitation, while the inclusion of a wider range of related infrastructure could triple the amount of investment required by 2030 [8]. Water management can greatly contribute to the development and implementation of sound public policies, with a shared responsibility between different levels of government, civil society, businesses, and the full range of stakeholders along with other decision makers who play an important role in achieving the environmental benefits of good water governance.

**Figure 1:**
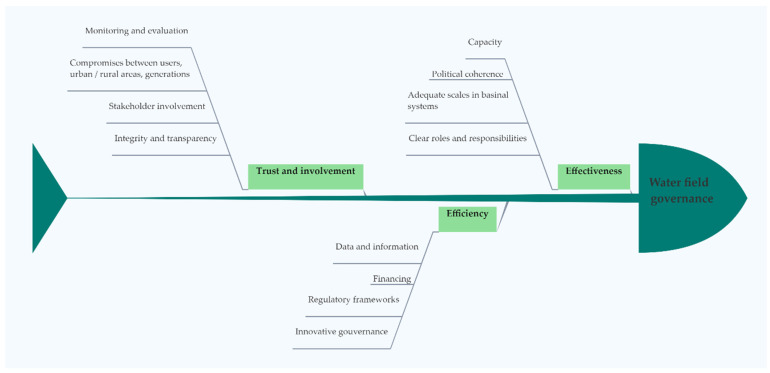
The OECD Principles on Water Governance [11] (Authors’ own source).

The OECD Principles on Water Governance intend to contribute to public policies that are results-oriented, based on three dimensions of consolidation, complementary to water governance, as shown in Figure 1 [11].

Therefore, effectiveness refers to the contribution of government, to clear objectives for sustainable water policy at all levels of government, and also to the implementation of these policy objectives and the achievement of those expected. Efficiency refers to the government’s contribution to maximizing the benefits of sustainable water management and well-being at the lowest cost to society. Trust and involvement are related to government contribution, but they also refer to strengthening the public trust and ensuring the inclusion of stakeholders through democratic legitimacy and equity for society as a whole [11,12]. Due to the many climate changes that people have been facing in recent years, both the intensity of droughts and floods is becoming an extremely important issue in terms of the quantity and quality of groundwater and surface water resources.

Moreover, the 2000s marked a promising beginning, or rather a resumption of European Union legislation, with a view to establishing a framework for community water policy. One of the key issues underlying the adoption of this directive was a Eurobarometer survey that was representative of all EU countries, in which citizens had to list five environmental issues that they were concerned about. The average results for the EU showed that almost half of respondents were concerned about “water pollution” (47%), with growth for each country up to 71%. That concern from citizens was one of the main reasons why the Commission made water protection one of the priorities of its work.

Overall, the Water Framework Directive 2000/60/EC (WFD) is arguably the most important directive on water, with the focus on environmental sustainability [13]. The European Commission is working closely with member states and stakeholders to better integrate the Water Framework Directive with other EU policies. Operational and rural development programs for the 2014–2020 period have been evaluated based on each program’s ability to measure its contribution to EU water policy. By highlighting the progress made so far, the resulting reports can contribute to improving future integration by [14]:

- assessing the contribution of operational programs to the implementation of EU water policy;
- providing key descriptive statistics on the consideration of water issues in rural development programs 2014–2020;
- developing guidelines for the development of a good practices from a water perspective.

Beyond these issues, the European Commission has launched a European Water Innovation Partnership (WIP) to support and facilitate the development of innovative solutions to water-related challenges and to create market opportunities in this regard. The WIP brings together all relevant stakeholders to identify priority areas for action, to identify barriers to innovation, and to propose solutions for eliminating future negative challenges. The European Economic and Social Committee (EESC) considers that “it is absolutely necessary to achieve the highest level of coordination of innovation processes at the European level, so that human and financial resources are used in the most efficient way possible to ensure innovation and to improve the population access to water resources and to use it more rational.” Innovation can make significant progress over time in terms of both water recirculation and how water is managed, so that water can be considered an abundant resource for future generations.

In Romania, there are a multitude of normative acts with information on the use and protection of this resource that is so important for a decent living. Article 35 of the Romanian Constitution evokes the right to a healthy environment, by the fact that “the state recognizes the right of every person to a healthy and ecologically balanced environment”, although the Constitution does make any strict reference to water resources. Unlike Romania, the right to water was introduced in Slovenia by its Constitution as a fundamental right for all citizens. “Water resources are a public good that is managed by the state. Water resources are used primarily and sustainably to provide citizens with drinking water and, in this sense, are not a commercial commodity,” one article stipulates [15].

In 2014, the Commission responded positively to a major EU citizens’ initiative. Specifically, the organizers of the Right 2 Water program requested that the European Commission ensure that all EU citizens have the right to water and sanitation, excluding water supply and water resources management from the rules of liberalization of the internal market and also stepping up efforts to ensure universal access to water and sanitation worldwide. Moreover, since 2014, the Commission has sought to identify shortcomings and areas where more needs to be done—at the community or at the national level—to address citizens’ concerns.

Thus, the European Commission has undertaken the following concrete steps and new actions in areas that are directly relevant to the initiative and its objectives [16]:

- step up efforts by member states to fully implement EU water legislation;
- launch a public consultation at EU level on the Drinking Water Directive to assess the need for improvements and how they could be achieved;
- improve citizen information by further developing efficient and more transparent management and dissemination of data on domestic and drinking water;
- explore the idea of benchmarking water quality;
- promote a structured dialogue between stakeholders on transparency in the water sector;
- cooperate with existing initiatives to provide a broader set of benchmarks for water services, improving the transparency and accountability of water service providers, giving citizens access to comparable data on key economic and quality indicators;
- stimulate innovative approaches to development assistance (e.g., support partnerships between water operators and public–private partnerships) and promote best practices between member states (e.g., on solidarity instruments);
- promote universal access to drinking water and sanitation as a priority area for the post-2015 sustainable development objectives;
- finally, call on member states, acting within the limits of their competences, to take into account the concerns of citizens through this initiative and to encourage them to step up their efforts to ensure safe, ecological, and accessible water supply for all.

According to Water.org, 663 million people do not currently have access to safe drinking water. According to a report by the Ellen MacArthur Foundation [17], the amount of plastic has doubled in the last 50 years, as PET production requires an investment of millions of tons of oil and 3 times more water to manufacture a single bottle than the amount of water that is actually contained in a single bottle. After a bottle’s use, it is discarded into nature, where the decomposition process begins only after 700 years. Unfortunately, recycling initiatives are quite trivial compared to the amount waste generated from the use of these PETs. Of course, an independent law referring exclusively to eco-innovation in the field of water would be welcome, especially since society is moving fast towards 2025, which statistically speaking, threatens us with diminished, even depleted resources on Earth.

Therefore, the complex problems arising from the lack of proper water management, but also with regard to water quality, require a transition to a kind of systemic thinking that can only be achieved through real transformation, with an emphasis on sustainable development and eco-innovation.

### 2.2. Romania Versus the EU Regarding General Indicators from the Eco-Innovation Field

**Figure 2:**
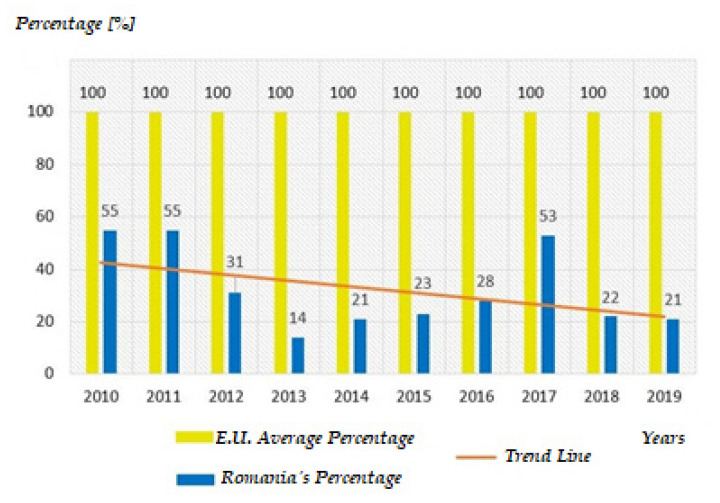
General indicators in the eco-innovation field versus Romania [10] (Authors’ own source).

From the point of view of general indicators in the eco-innovation field, in relation to the average of EU states, Romania registers minimum values in this sector. Romania’s performance can be seen in the graph from Figure 2 [10,18].

The maximum percentage of 100% represents the apogee of fulfilling the indicators in the field of eco-innovation, at the level of the European Union. Meanwhile, the average of the member states registers a high level in this respect; unfortunately, Romania over the last decade has registered a decline below the average value when it comes to fulfilling these indicators.

The scores indicated in Figure 2 were calculated based on existing data and were published by “Eco-Innovation Scoreboard Interactive tool” [6]. The E.U. score average represents the weighted average of the scores obtained by the 29 countries included in the analysis during the course of the decade 2010–2019, while Romania’s score represents the values obtained annually by this country during the analyzed period. Although the annual scores are variable in Romania, the trend line shows a decreasing trend of the level of compliance with the general indicators of eco-innovation.

In the graph from Figure 2, it can be seen that unlike the average of the European Union member states that accumulates a score of 100 points in eco-innovation, Romania registered in the period 2010–2017 values below average in all important stages: between 2010–2011, Romania recorded the same total score of 50 points as it had in 2017; in the period 2011–2013 Romania had a drastic decrease, so that it ended up accumulating a score of only 25 points so that from 2014 to 2017 Romania seemed to record exponential increases, although its resulting scores were still below the average of EU countries. Therefore, the country’s performance cannot be spoken of as a significant and progressive evolution when referring to eco-innovation in Romania. The reasons are many, but it is clear that the private sector needs sufficient resources to build technology and know-how specific to the development of this sustainable industry and the consumption of its output.

A high degree of eco-innovation entails certain benefits for industry operators: accessing new and emerging markets; increasing profitability along the value chain of products; attracting investors and implicitly investments; increasing the productivity and technical capacity of products, strengthening the organizational capacity for collaboration and exchanging information between companies, and optimizing the use of transport and materials, resulting in cumulative savings in operational efficiency, etc. [19].

The effects of using drinking water from public networks has a direct influence on the population, more precisely on their state of health. Most of the time, in order to not get sick, citizens choose, or in other words feel compelled, to consume bottled water, thus indirectly supporting the manufacture of polyethylene terephthalate (PET) and also indirectly having a role in the waste of water used for PET manufacture, beyond the contents of the bottle. Therefore, the industry is witnessing a whole vicious circle that has severe repercussions both on the health of the population and on the environment, which in turn also takes revenge on the human factor.

### 2.3. Eco-Innovation, a Mechanism towards Sustainability

Sustainability is an independent concept that Lester Brown, the founder of the Worldwatch Institute, defined in 1981 as the “support capacity” of resources to carry out any activity in an organization [20]. The difference between the concept of sustainable development and sustainability lies in the fact that the first refers to the overall development of a country from an economic point of view, while the second points to the existing development aspects within some enterprises. Therefore, sustainability is related to economic, environmental, and social performance [21,22,23]. One of the mechanisms of sustainability in enterprises is eco-innovation, presented as a model of evolution in the socio-economic field.

For enterprises, eco-innovation is seen as a mechanism for reducing the negative impact on the environment caused by the enterprises themselves.

**Figure 3:**
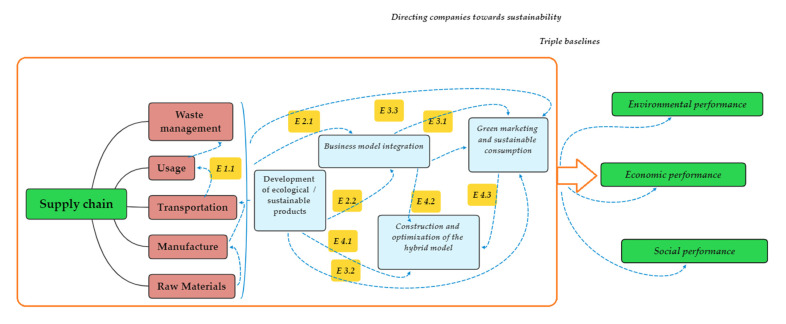
Evolution of companies over time towards sustainability [24] (Authors’ own source).

Figure 3 [24] shows from a technological point of view the evolution of companies over time towards sustainability, as well as the measures they have taken over time to reach this goal that is so coveted by the actors in the socio-economic environment.

Figure 3 shows that at the E1 stage, companies are encouraged to develop environmentally friendly and sustainable products, based on the concept of extended producer responsibility; however, this aspect cannot be developed unilaterally. Therefore, in phase E2, the members of the supply chain companies are also incorporated, together, in order to achieve sustainability, which generates a “closed loop supply chain” (E2.1). Moreover, supply chain optimization and risk analysis are also considered. Therefore, innovative business models are introduced and developed (E2.2). In stage 2, although ecological/sustainable products and business models are developed, the companies find that their customers are not loyal to their products. Therefore, in stage 3, a project for sustainable consumption is developed (E3.1, E3.2). On the other hand, with the development of the closed supply chain, recycling aspects are promoted. Moreover, proper waste management is also taken into account (E3.3). In stage 4, new technologies are developed with more responsibility. Businesses need to build an optimized hybrid model (E4.1, E4.2, and E4.3) [24].

Over time, corporate sustainability has become of increasing interest as a result of the Brundtland Report, which in 1987 spoke of the importance of green technologies [25]. Eco-innovation can be found in several areas [26]:

1. Environmental technologies—comprising technologies used to limit and control the pollution emitted and technologies that limit the use of resources and materials in production;
2. Organizational innovation—a new environmental management based on the concept of life cycle assessment as well as on cooperation between organizations;
3. Innovation of products and services—including changes in the design and generation of products or services;
4. Green system innovation—the use of alternative production and consumption systems, more environmentally friendly than those used so far.

Decades have passed from the theoretical aspects of eco-innovation to its implementation, so that now the practical barriers that have arisen as a result of the desire to implement eco-innovation in enterprises, as well as about the benefits that it brings later can be talked about more specifically. Each type of activity sector experiences barriers to the implementation of eco-innovation. Among these, the most common issues faced by businesses are the lack of financial resources, high implementation costs, reluctance to recover money invested in eco-innovation or the existence of an investment recovery period that is much too long, difficulties in finding an economic partner, reluctance to risk, lack of qualified human resources, as well as lack of knowledge on the implementation of eco-innovation.

The most important benefits of implementing eco-innovation in enterprises relate to reducing production costs, increasing sales, as well as improving services, not to mention knowing and complying with increasingly restrictive legal requirements on environmental protection.

## 3. Materials and Methods Used in the Research

The present research started from our analysis of the eco-innovation field in drinking water management as a path to sustainability and by implementing improvements such as the circular economy instead of a linear economy. The activities carried out within the sub-objectives that led to the achievement of the main desideratum assumed on the one hand identification of all drinking water suppliers in the underdeveloped regions of Romania, analysis of all existing and published statistical data on their activity, and evaluation of the activity of these operators based on specific indicators in this field.

This indicators were extracted from a more comprehensive group of indicators established at the level of the European Union, which although the indicators are described quite vaguely in the existing documentation at European level, based on the name of these indicators, an extremely suggestive reference is made in the sense of what could be evaluated by using them (they allowed the analysis of the degree of eco-innovation and sustainability in drinking water management, reflecting a classification of the results of technical, economic, and environmental activities but also under internal legal aspects—regarding the legal restrictions in Romania).

On the other hand, based on the study conducted using the chosen indicators, by applying a scoring system, it was possible to achieve a centralized image of the degree to which eco-innovation measures among the analyzed drinking water supply companies were implemented, and then by using methods of statistical analysis, we achieved a clear classification of analyzed companies positions and delimitation into classes that suggested either a high, medium, or low level of eco-innovation implementation in the field of drinking water supply. The analyses carried out were based on the hope of encouraging users to increase the amount of drinking water consumed from suppliers who had implemented a medium to high level of eco-innovation and therefore suppliers who provided drinking water with superior qualities (while discouraging the use of bottled drinking water in PET packaging), but also the analyses highlighted companies that had a low degree of eco-innovation in the field of drinking water, with results that represent clear evidence of an urgent need to improve the activity, both within the operation of the companies and especially through policies and administrative support.

### 3.1. Description of the Research and Specific Objectives Pursued

The core of the effectiveness research was to identify the degree of eco-innovation among drinkable water providers from the seven development regions of Romania. The results could thus reflect a global image of this perspective in Romania and, most important, could also give a solid starting point for considering policy decisions regarding how to invest in or otherwise support the regions with poorer performance (in terms of help by equipping or improving existing sewage treatment plants, by providing technological improvements through competitive equipment, and last but not least, by replacing drinking water supply pipes, as the age of the distribution network and their wear is an important factor in achieving the proposed goal).

This stage of the research was aimed at determining the economic operators influencing the degree of eco-innovation in relation to household consumers, as well as the degree of encouraging more efficient and responsible consumption of water. The research was qualitative in nature, the data provided were from an online field survey, and the investigation technique was performed in the form of a case study.

The study was conducted based on existing public data on the websites of drinking water supply companies as of 2020. The research approach was chosen because of the need for companies to take measures to improve eco-innovation in important sectors such as drinking water as a result of the general analysis of eco-innovation indicators observance in Romania in the decade between 2011 and 2019.

The target group consisted of regional economic operators in Romania that supply drinking water. Depending on the statistical results obtained, based on indicators, a better and more sustainable consumption method was chosen, both for the environment and for the citizens.

The main activities carried out that led to the achievement of the proposed objective were:

1. Identification of economic operators—drinking water suppliers in Romania;
2. Identification and establishment of indicators used in the evaluation of economic operators;
3. Realization of a value scale based on the figures obtained by each economic operator in the field of eco-innovation;
4. Implementation of a comparative analysis between the economic operators, based on the established indicators;
5. Interpretation of final results.

#### Identification of Economic Operators—Drinking Water Suppliers in Romania

A key question to ask was what it means to be a regional economic operator in this context. Briefly, it can be said that a regional economic operator is the one that manages and operates the water supply and sewerage system in an administrative-territorial unit [27].

In this sense, on the National Regulatory Authority for Community Services of Public Utilities (ANRSC) website, the regional economic operators—drinking water suppliers from Romania—were identified [28]. The names of the analyzed operators were coded in accordance with the data presented in the next table.

These data can be found in Table A1 (indexed in Appendix A):

Table A1 shows a total number of 46 enterprises that supply drinking water to Romanian citizens and that operate according to Law 241/2006 on the water supply and sewerage service [29].

### 3.2. Identifying and Establishing the Indicators Used in the Evaluation of Economic Operators

**Figure A1:**
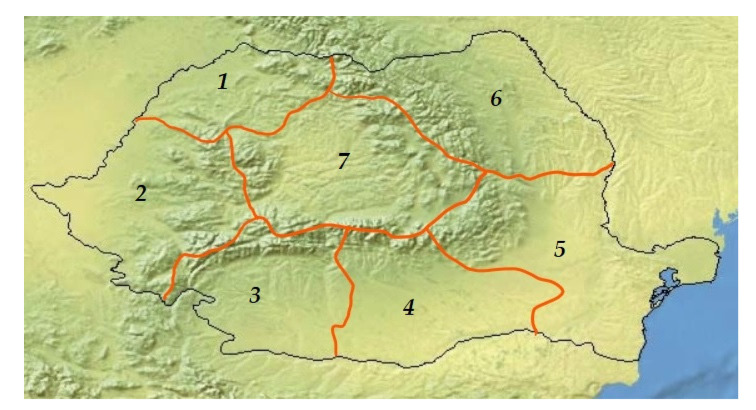
The seven development regions of Romania [30].

For a unitary approach, the most important cities with water treatment plants located at the same level of development, from the 7 development regions of Romania (graphically represented in Figure A1 of the Appendix A [30]), were taken into account, as follows:

1. From Region 1 (Northwest Region)—Satu Mare, Cluj-Napoca, Oradea;
2. From Region 2 (West Region)—Timișoara, Arad;
3. From Region 3 (Southwest Region)—Craiova;
4. From Region 4 (South Region)—București, Ploiești, Pitești;
5. From Region 5 (Southeast Region)—Galați, Tulcea;
6. From Region 7 (Northeast Region)—Iași, Suceava;
7. From Region 8 (Center Region)—Alba Iulia, Sibiu, Brașov.

Since an indicator can be a tool that is useful for quantifying in equal measure quantities, phenomena, and information, the use of the indicators technique was of great interest in assessing the level of eco-innovation of drinking water providers from Romania [31].

In another more technical definition, indicators are seen as “the result of an evolution over time, suggesting dynamics against a baseline, with or without a strategic purpose” [32].

The European Parliament called on the Commission to develop indicators on resource efficiency in order to monitor progress towards the circular economy. On 16 January 2018, the European Commission adopted a monitoring framework for the circular economy, consisting by a set of key and significant indicators.

According to the Eco-Innovation Observatory, which is an initiative funded since 2010 by the European Commission through the Directorate-General for the Environment under the Competitiveness and Innovation Framework Program, Eco-IS was developed as a tool for assessing and illustrating the performance of eco-innovation in EU member states.

At the moment, there is no single universally recognized indicator of eco-innovation and the circular economy, which is why there are currently few conclusive indicators that have been developed to describe the most relevant trends. By a single measure or with a single result, it would not be possible to uncover the many dimensions influencing the transition from a linear to a circular economy.

Therefore, the Eco-Innovation Scoreboard includes 16 indicators grouped into 5 important categories: eco-innovation inputs, eco-innovation activities, eco-innovation outcomes, resource efficiency, and socio-economic outcomes [33].

From the total number of indicators included in this study, the most relevant ones for economic operators were selected—drinking water suppliers, along with other indicators considered useful for research.

Therefore, our analysis resulted in 9 specific indicators belonging to 6 main groups, which were derived from the General Key Indicators adopted by the EU through its monitoring framework for the circular economy [34]. The main feature of these indicators was that they could be used for the analysis of eco-innovation management in all drinking water supply companies from the underdeveloped regions of Romania.

**Figure 4:**
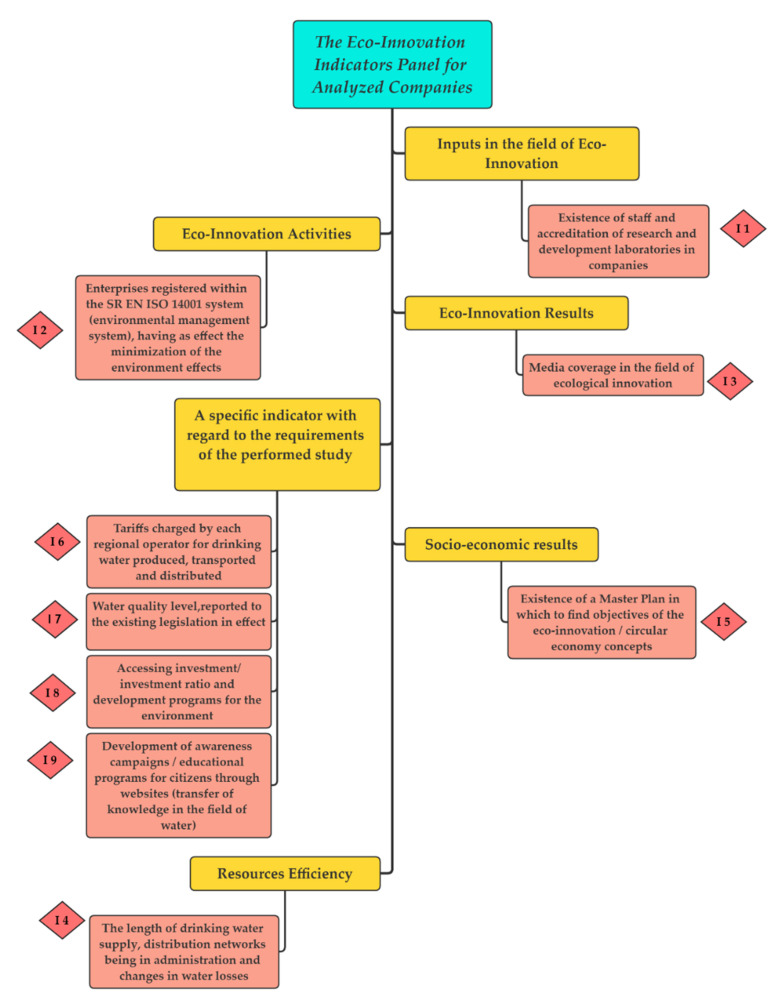
Eco-Innovation Indicators by categories (Authors’ own source).

For a better understanding of the impact on the performed study gained by the indicators (I) presented in Figure 4, they are described in Table 1 (in the description below regarding the way points were awarded, the symbol “p” represents—“points”, being therefore the unit of measurement for the score values).

It should be noted that each criterion had an equal weight in terms of meeting the specific requirements of eco-innovation. Therefore, the nine indicators had the same degree of importance in the evaluation of operators.

### 3.3. Case Study on Performing a Comparative Analysis between Economic Operators

Eco-innovation is an important pawn in the entrepreneurial environment, particularly because it helps to increase the competitiveness of regional operators. CNPCD management expresses the fact that according to European statistics, in our country, only half of the population has access to drinking water, and the negative aspects of this, as well as others negative aspects in the field of environment, can be stopped with the help of eco-innovation [36].

The four most important benefits are those aimed at the economic sector—by creating new business models derived from the circular economy; the environmental sector—through an adequate and sustainable management of natural resources in parallel with combating climate change; in the sphere of society—by creating more jobs; but also in the political sphere, by allocating resources fairly [37,38].

The nine indicators used in the analysis were those previously described in Section 3.2 and are found in Figure 4 (I1–I9). There, these indicators were structured so that they would be the most conclusive for the research conducted (according to the monitoring framework for the circular economy), being able to offer perspectives in several directions and providing a plurality of measures and results in the sense of improving eco-innovation and supporting the circular economy among drinking water suppliers.

The research methodology consisted of evaluating each regional economic operator, as shown in Table 2, using a scale from 1 to 5 for each indicator and ranking them according to the score obtained.

The final evaluation scale was in accordance with the following values:

- Operators with high performance in eco-innovation: 4–5 points;
- Operators with average performance in eco-innovation who had to improve certain aspects: 2–3 points;
- Operators with poor performance in eco-innovation who meet the minimum criteria: 0–1 points;

In order to make it possible to draw a pareto diagram that allowed the classification of companies according to the scores obtained based on the criteria related to eco-innovation indicators, the relative cumulative frequency of eco-innovation scores was calculated using Equation (1):

(1) $$\mathrm{Fc}r_{i}=\left(\frac{\mathrm{Vc}f_{i}+\sum_{i=1}^{i-1}Vcf_{i}}{Tp}\right)*100$$

where V_cfi_ is the current value of eco-innovation scores, and T_p_ is the total eco-innovation scores.

## 4. Results and Discussion

In accordance with the evaluation methodology of the regional operators, using the scores of the points awarded in relation to the acceptance of the established eco-innovation indicators, the obtained results are presented in Table 2.

Therefore, from the results of the analyses carried out, it can be observed that the regions are arranged according to the total number of points gained, reflecting a decrease in region scores from the first region presented in the table to the seventh region presented. The score obtained by each region analyzed is composed of the sum of the number of points granted to each regional operator included as part of that region. The score granted was obtained in relation to the final evaluation scale among the operators from that region, which contained a different number of points for a high, average, or poor performance in the eco-innovation field. This level of performance was established based on the 9 indicators already presented.

From the analyzed data, some important aspects can be disseminated:

(1) As a result of the synthesis and structuring of information on the eco-innovative analysis for the regional operators’ activity, it was possible to obtain graphical results structured by development regions, regional operators, and eco-innovation indicators.

The graphic interpretations of the research results performed allow an overview of the details set out in Table 2, thus giving the possibility of a quick identification of the classified regions according to the specified ranking of each. This allows an easy identification of the lowest ranked regions and leads to an introspection on the causes that made those low ranks possible. Here, it refers to the identification of specific companies in the respective regions that need to take more substantive eco-innovative improvement measures. Further, that identification was possible by developing two Pareto analyses of the companies involved and of the indicators linked to their activity.

**Figure 5:**
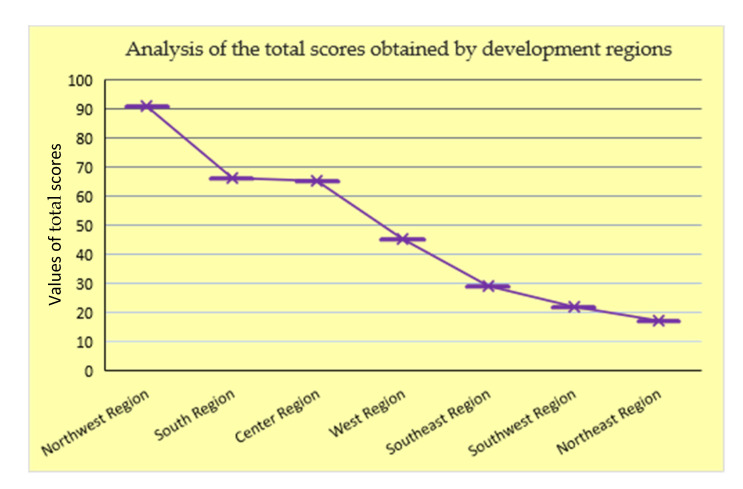
Graphical distribution of the total scores obtained by the regions analyzed (Authors’ own source).

The results of the analysis are presented in the graph in Figure 5. We thus found that the scores could be divided into three categories:

- The category of high scores, obtained by the Northwest Region;
- The category of average scores, which included the Southern, Center, and Western Regions;
- The category of minimum scores, in which fell the Southeast, Southwest and Northeast Regions.

**Figure 6:**
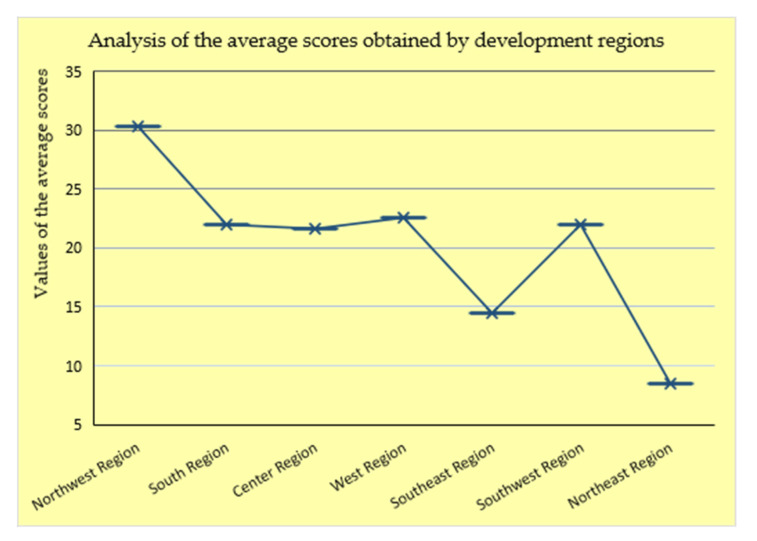
Graphical distribution of the average scores obtained by the regions analyzed (Authors’ own source).

Additionally, taking into account the fact that the analyzed regions consist of either two, three, or four companies each, for a unitary approach as a result of the eco-learning criteria evaluation, an analysis of the average scores obtained by the regional operators was carried out, which was related to the development regions from which they came. The results of the analysis are shown in the graph in Figure 6.

Therefore, from the graph shown in Figure 6, presents the average of the scores obtained by the regions in descending order. According to the total score obtained (the same order as in Figure 5), there is a decrease relatively equal to that identified among the total scores, with one exception in the right for the Southwest Region. This exception comes from the fact that in that region only one city could be considered (the largest zonal center that is the source of drinking water distribution) and compared with any of the other regions analyzed.

Therefore, in the first two positions are the Northwestern and Western regions, with scores of 33.3 points and 22.5 points, respectively. The next two regions—Southwest and South—have equal values of 22 points. In the last three positions are found the Center region, with a score of 21.6 points, the Southeast with a score of 14.5 points, and in the last position, with a score of only 8.5 points is the Northeast region. From the two graphs, as a result of the scores accumulated by each region, it can be concluded that the highest eco-innovation rate was recorded in the Northwest region, corresponding to the following 3 regional water service operators: C 38 A.S.M., C 14 C.A.S.C.N., and C 5 C.A.O. Next, for an intrinsic study of the performance of regional operators, a Pareto analysis was carried out, which identified the main 20% of operators that met 80% of the requirements imposed by the established performance indicators. To obtain the Pareto distribution, the following steps were followed [39]:

➢ Descending ordering of the analyzed elements (distribution of regional operators’ scores based on established indicators);
➢ Calculation of the relative cumulative frequency of eco-innovation scores obtained by the regional operators;
➢ Graphic tracing of the columns corresponding to each analyzed element (score value);

Identification of the 20% regional operators that met the highest number of scores related to eco-innovation was based on the intersection of the 80% threshold with the variation curve of the relative cumulative frequency of the scores obtained.

**Figure 7:**
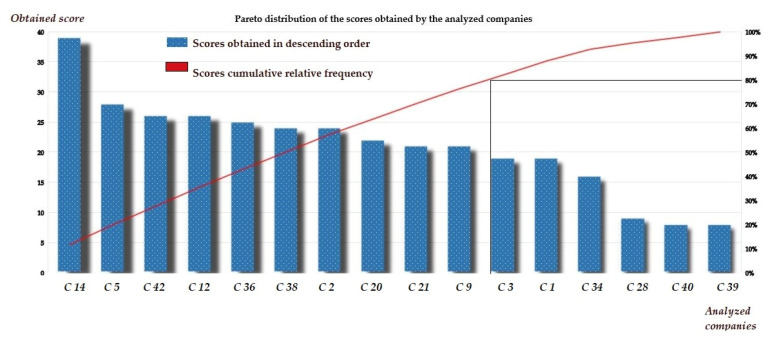
Pareto distribution of the relative cumulative frequency of the scores (Authors’ own source).

The result of the Pareto analysis is presented in Figure 7.

Based on the scores obtained, 20% of the top 10 regional operators met 80% of the eco-innovation requirements. At the same time, the most eco-innovative companies, in view of the high values of the scores obtained, were C 14 C.A.S.C.N., followed by C 5 C.A.O., and in third place with equal points, C 42 S.A.T. and C 12 A.N.B. (26 points each).

Regarding the study of eco-innovation indicators, following the steps described above, a Pareto ABC analysis was performed that allowed the indicators to be classified into 3 categories (A, B, C) according to the scores assigned to each indicator, in terms of its importance reflected by its implementation among regional operators.

**Figure 8:**
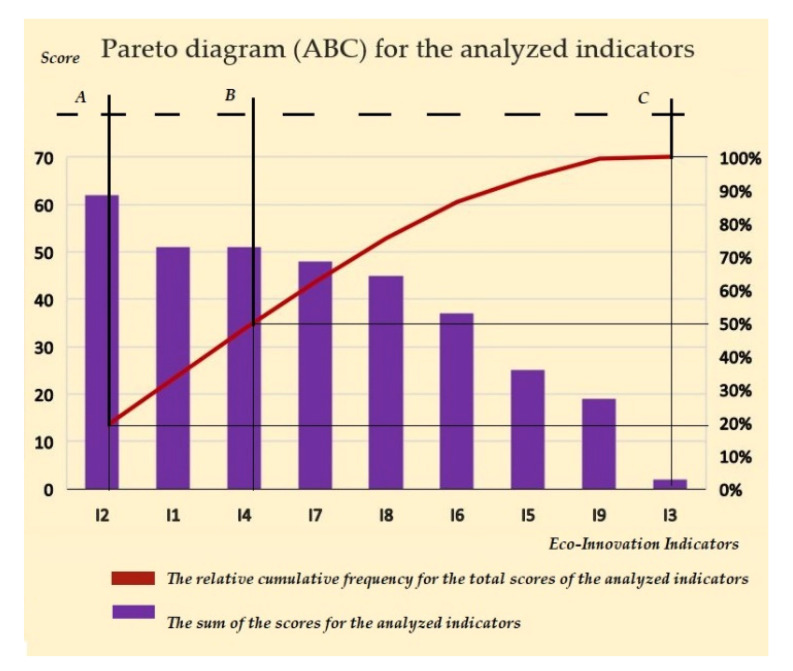
Pareto ABC diagram of indicators (Authors’ own source).

The result obtained is presented in Figure 8.

- Category “A” comprises 20% of the total scores assigned to eco-innovation indicators, consisting of the first indicator analyzed (I 2), which is thus the most respected criterion by regional operators and is therefore the most important one.
- Category “B” comprises 30% of the total scores assigned to eco-innovation indicators, consisting of 2 indicators (I 1 and I 4), these having random occurrences and medium influence in the order of importance for compliance by regional operators.
- Category “C” comprises 50% of the total scores assigned to eco-innovation indicators, consisting of the other 6 indicators, which have a low incidence rate among regional operators.

## 5. Conclusions

An essential aspect of this research is the belief that drinking water must be protected and used in the most efficient and sustainable way for the benefit of the population.

In this way, a study was undertaken to analyze the eco-innovation level of economic operators responsible for providing drinking water to the country’s citizens, based on nine indicators. The analysis performed resulted in the identification of the most respected indicators based on the scores awarded, which were I 2, measuring enterprises registered within the SR EN ISO 14001 System, and I 7, measuring water quality level reported in compliance with the existing legislation that is in effect. The least implemented indicators were I 3, assessing the extent of media coverage in the field of ecological innovation, and I 9, a measure of awareness campaigns/educational programs developed for citizens through websites.

In the case of regional operators, following the evaluation, an acute lack of eco-innovation implementation in companies was found, with the highest scores in the country, based on the indicators used, being obtained by companies C 14 C.A.S.C.N., C 5 C.A.O., C 42 S.A.T., and C 12 A.N.B. The low presence of eco-innovation in drinking water distribution companies can lead, among other things, to the low interest (often reflected by lower end-of-network drinking water quality) of citizens in drinking water from the public network, which may lead the population towards the use of bottled drinking water in PET bottles.

Based on the research conducted, the results show that policy attention mostly needs to be focused on underpinning the efforts of drinkable water providers through financial investments and legal support, especially in the regions least developed in the sense of eco-innovation, such as the Northeast Region, Southeast Region, and Center Region. This can be done through measures that allow improvements in the technological performance of the suppliers and of the treatment plants (or the realization of such stations where they do not exist) as well as through the replacement of some sectors of drinking water distribution networks, where required. Taking into account the specific measures based on the indicators underlying the study, eco-innovation is reflected by the performance of state-of-the-art equipment that is environmentally friendly, which also helps to analyze the cost of service. As it has already been found, if some companies are obliged to use a certain type of equipment in a new water treatment plant, other existing companies must receive financial support for similar refurbishment to become a priority. This support can come from administrative channels. On the other hand, the mandatory extension or replacement of used or old distribution networks could become a priority for the responsible authorities in order to encourage the application of sustainable eco-innovation in drinking water management. These urgently needed actions can be supported both nationally and regionally, locally or even by the companies that perform and provide these services. The immediate results of taking such measures will be highlighted by raising the degree of compliance with eco-innovation indicators in this field that have been imposed at the European level. Internationally, unlike the average of the European Union member states that accumulate a score of 100 points in eco-innovation, Romania registered values below average in every year of the 2010–2017 period, which is an important sign that expresses the fact that Romania is not yet ready to move towards an eco-innovation plan.

## Figures and Tables

**Table 1:** Description of the eco-innovation indicators used.

Indicator Code	Indicator Description
I 1	The fact that there are qualified internal staff and properly equipped research laboratories implied the possibility of continuous monitoring of the quality of the drinking water supplied, but through regular tests and process improvement, based on the knowledge of research staff and innovative methods, and starting from the need for permanent issues faced by the supplier, it was also a measure of the quality of the production process itself. Accreditation of laboratories is performed by the Romanian Accreditation Association (RENAR); the classification was made according to the number of accredited units [34]. For the maximum number of units accredited into a region = 5 p, for a medium number = 3 p, and for a minimum number = 0 p.
I 2	Ensured the implementation of a quality and/or environmental management system (ISO type) certifies compliance with the minimum legal norms (especially ensuring minimal impact on the environment) and allows the identification of the quality level of services and product provided by the distributor. For companies that implemented an integrated management system = 5 p, for companies that implemented only ISO 9001 standard = 3 p, and for companies that did not declare their certification = 0 p.
I 3	Allowed analysis of the results of the company’s promotion activities on increasing its impact on users, both for raising their number, but especially for the quantity of product used by them. For companies that were involved in various promotion campaigns (such as socio-marketing or exposing the refurbishment, presenting the laboratory results or questioning the consumer public) in addition to using several means of mass communication = 5 p, for companies that limited their communication with consumers (formal communication by publishing general data) = 3 p, and among companies disinterested in communication and constantly updating data of public interest = 0 p.
I 4	Allowed the evaluation of the degree of implementation of eco-innovative measures at the drinking water production station level in relation to the area of the drinking water distribution network in order to ensure a more efficient management of resources in terms of their optimization and the rational operation of this distribution network. This indicator covers the entire existing kilometers of water distribution networks of regional operators. Additionally, it covers the changes in water losses from the entire network. Therefore, it does not refer exclusively to cities but refers instead to the entire communities of operators. Operators that registered high performance compared with the established indicator had to accumulate a minimum length of water distribution networks of 1000 km (≥1600 = 5 p); those with average performance were in the length of at least 700–1000 km (>800 = 3 p); low-performance operators fell into the 200–700 km length category (≥200 = 1 p).
I 5	This indicator involved identifying the company’s level of interest in continuous development through the existence of a Master Plan that takes an eco-innovative direction and has socio-economic impact by improving technological equipment, by qualifying and continuously enriching workers’ knowledge, and by employing measures to support ecological and economic efficiency and optics of orientation towards the circular economy, etc. The tie-breaking criterion consisted of the number of objectives specified in the Master Plan for each regional operator: between 6–8 objectives (≥8 = 5 p); 3–5 objectives (>4 = 3 p); 0–2 objectives (≥1 = 1 p).
I 6	This indicator allowed the identification of the pricing regulation level of the product provided in relation to the services offered in order to encourage the large-scale use of drinking water distributed to the population. The price in money/m^3^ was taken into account. The tariff established for granting the score in accordance with this criterion was represented by the value of the drinking water supply cost expressed in the national currency (RON) related to a volume of 1 m3. The reported price was in accordance with the one declared by the suppliers with the interval between 2.5 and 4 RON on a unit volume of water. The operators that registered high performances compared with the established indicator had to register the following price values: ≤2.85 L/m^3^ = 5 p; ≤3.44 L/m^3^ … x … ≥3.30 l/m^3^ = 3 p; ≥3.78 = 1 p. The tariffs approved and practiced by each regional operator can be found on the National Regulatory Authority for Community Public Utilities Services (ANRSC) website [35]. This indicator was important mainly because all the consumers need to have access to water at a lower price. If this indicator was skipped, consumers would therefore try to buy bottled water, which sometimes is cheaper, but PET waste production would increase.
I 7	By using this indicator, the quality level of drinking water provided by operators could be clearly identified by comparing the values of physical-chemical parameters of drinking water, measured in situ and relating those values to the values imposed by current legislation. For this indicator, physico-chemical parameters were taken into account (mainly the following were taken into account: turbidity, pH, electrical conductivity, hardness, free residual chlorine, ammonium, nitrogen, lead). For the evaluation of operators, the number of non-conformities related to water analyzes at the points chosen for sampling were taken into account. Operators that recorded high performance compared with the established indicator had to have recorded the following values: 0 non-conformities = 5 p; between 1 and 3 non-conformities = 3 p; >3 = 1.
I 8	This indicator also contains investment ratio, which allowed the analysis of the degree of interest in fulfilling the company’s objectives in relation to the need to take measures to improve environmental performance, both through internal actions and by requesting political support through administrative involvement. The indicator of investment and development programs for the environment aimed to highlight the activity of attracting European funds and of involving regional economic operators in the area of environmental protection and modernization in which they carry out their economic activity. In this sense, the degree of access to projects from European funds was pursued with an exclusive focus on the Large Infrastructure Operational Program (POIM) and Sectoral Operational Program (POS)-Environment programs. The evaluation and tie-breaking criteria were as follows: Operators who accessed both environmental programs = 5 p; Operators who accessed single program = 3 p; Operators who did not access any program = 0 p.
I 9	This indicator assessed the degree of communication between supplier and user, specifically communication that had as its special purpose raising users’ awareness of the importance of supporting the environment by using the drinking water provided, communication based on clear evidence of the drinking water quality, and communication following the implementation of a compendium of measures for continuous improvement of the activity and of the obtained and provided product. This indicator aimed to observe the degree of involvement of regional operators in educating and informing citizens, as well as capturing their relationship with the social environment in terms of care for that environment. Depending on the number of the operator’s initiatives, the following classification of the given score was performed: Operators who had more than 2 initiatives: 5 p; Operators who had a single initiative = 3 p; Operators who had no initiatives = 0 p.

**Table 2:** Evaluation of regional economic operators.

Indicators		I 1	I 2	I 3	I 4	I 5	I 6	I 7	I 8	I 9	Total Score of Each Company
**Awarded Score (Between Min 0–Max 5 points)**											
**1. Northwest Region**											
**City/Company**	C 38 A.S.M	**3**	**5**	**0**	**3**	**0**	**3**	**5**	**5**	**0**	**24 points**
	C 14 C.A.S.C.N.	**5**	**5**	**0**	**5**	**5**	**4**	**5**	**5**	**5**	**39 points**
	C 5 C.A.O	**5**	**5**	**0**	**4**	**3**	**3**	**5**	**3**	**0**	**28 points**
**The total region score of 91 points**											
**The average region score of 30.33 points**											
**2. West Region**											
**City/Company**	C 42 S.A.T.	**3**	**5**	**0**	**5**	**0**	**5**	**5**	**3**	**0**	**26 points**
	C 3 C.A.A.	**3**	**5**	**0**	**4**	**0**	**2**	**5**	**0**	**0**	**19 points**
**The total region score of 45 points**											
**The average region score of 22.5 points**											
**3. Southwest Region**											
**City/Company**	C 20 C.A.O.	**3**	**5**	**0**	**1**	**3**	**2**	**5**	**3**	**0**	**22 points**
**The total region score of 22 points**											
**The average region score of 22 points**											
**4. South Region**											
**City/Company**	C 12 A.N.B	**3**	**5**	**0**	**5**	**1**	**2**	**5**	**0**	**5**	**26 points**
	C 35 H.P.P.	**0**	**2**	**0**	**5**	**3**	**1**	**5**	**0**	**0**	**16 points**
	C 2 A.C. 2000	**5**	**5**	**2**	**3**	**1**	**2**	**3**	**0**	**3**	**24 points**
**The total region score of 66 points**											
**The average region score of 22 points**											
**5 Southeast Region**											
**City/Company**	C 21 A.C.G.	**3**	**5**	**0**	**1**	**3**	**1**	**0**	**5**	**3**	**21 points**
	C 40 A.T.	**0**	**0**	**0**	**1**	**1**	**1**	**0**	**5**	**0**	**8 points**
**The total region score of 29 points**											
**The average region score of 14.5 points**											
**6. Northeast Region**											
**City/Company**	C 28 A.I.	**3**	**0**	**0**	**4**	**0**	**2**	**0**	**0**	**0**	**9 points**
	C 39 A.S.	**0**	**5**	**0**	**1**	**1**	**1**	**0**	**0**	**0**	**8 points**
**The total region score of 17 points**											
**The average region score of 8.5 points**											
**7. Center Region**											
**City/Company**	C 1 C.T.T.A.	**5**	**5**	**0**	**2**	**1**	**3**	**0**	**3**	**0**	**19 points**
	C 36 A.C.S.	**5**	**0**	**0**	**5**	**3**	**4**	**5**	**3**	**0**	**25 points**
	C 9 C.A.B.	**5**	**5**	**0**	**2**	**0**	**1**	**0**	**5**	**3**	**21 points**
**The total region score of 65 points**											
**The average region score of 21.6 points**											

In the Table 2, the legend of colors used for scaling scores represents: green, maximum value of 5 points; blue, 4 points; dark grey, 3 points; light grey, 2 points; red, 0 points; and orange, used for region categories.

## Data Availability

Data sharing not applicable.

## Appendix A

**Table A1:** Coded name of the analyzed companies.

No.	Operator Code Name
1.	C 1 C.T.T.A.
2.	C 2 A.C. 2000
3.	C 3 C.A.A.
4.	C 4 C.R.A.B.
5.	C 5 C.A.O
6.	C 6 A.C.N.V.
7.	C 7 A.B.N.
8.	C 8 C.U.P.D.
9.	C 9 C.A.B.
10.	C 10 N.A.B
11.	C 11 C.D.A.B.
12.	C 12 A.N.B
13.	C 13 E.C.
14.	C 14 C.A.S.C.N.
15.	C 15 C.A.A.T.
16.	C 16 A.C.R.
17.	C 17 R.C.
18.	C 18 G.C.S.G.
19.	C 19 C.A.T.D.
20.	C 20 C.A.O.
21.	C 21 A.C.G.
22.	C 22 A.S.G.
23.	C 23 A.P.D.
24.	C 24 A.S.V.J.
25.	C 25 H.M.C.
26.	C 26 A.C.I.
27.	C 27 E.A.V.
28.	C 28 A.I.
29.	C 29 S.D.T.S.
30.	C 30 V.B.M.
31.	C 31 C.A.T.M.
32.	C 32 C.J.A.S.N.
33.	C 33 C.A.O.S.
34.	C 34 A.N.P.
35.	C 35 H.P.P.
36.	C 36 A.C.S.
37.	C 37 A.T.M.M.
38.	C 38 A.S.M.
39.	C 39 A.S.
40.	C 40 A.T.
41.	C 41 A.S.A.
42.	C 42 S.A.T.
43.	C 43 A.R.V.
44.	C 44 A.C.T.J.
45.	C 45 C.U.P.F.
46.	C 46 A.V.
